# Skin Cancer Knowledge, Attitudes, and Behaviors in Collegiate Athletes

**DOI:** 10.1155/2014/248198

**Published:** 2014-03-24

**Authors:** Courtney Hobbs, Vinayak K. Nahar, M. Allison Ford, Martha A. Bass, Robert T. Brodell

**Affiliations:** ^1^Athletic Department, Southern Methodist University, 5800 Ownby Drive, Dallas, TX 75275, USA; ^2^Department of Health, Exercise Science & Recreation Management, The University of Mississippi, 215 Turner Center, P.O. Box 1848, University, MS 38677, USA; ^3^Department of Dermatology, University of Mississippi Medical Center, UP Pavilion, Suite K, 2500 N., State Street, Jackson, MS 39216, USA

## Abstract

Outdoor athletes represent an important group at risk for skin cancer because they are routinely exposed to high levels of ultraviolet radiation. The purpose of this study was to assess current skin cancer knowledge, attitudes, and behaviors among collegiate athletes. A modified version of the Melanoma Risk Behavior Survey was completed by 343 athletes attending a Southern University in the USA, generating an 87% response rate. Survey results demonstrated that the majority of the athletes do not limit their sun exposure and reported low levels of sun protective behaviors. In addition, athletes lacked knowledge about skin cancer and sun protection. Eighty-three percent of the athletes stated that tanning beds improve one's overall health. Race was significantly associated with skin cancer knowledge, whereas, gender was found to be significantly associated with knowledge, attitudes, and behaviors towards skin cancer. Additionally, there was a significant relationship between knowledge and behavior, but not between attitude and behavior. This study highlights the need to educate athletes about the hazards of tanning to minimize UV exposure and promote sun protection habits. Moreover, athletes should be educated on the dangers of indoor tanning facilities and encouraged to avoid these facilities.

## 1. Introduction

Skin cancer is the most commonly diagnosed cancer in the USA, with over 3.5 million cases recorded per year [1]. While basal cell carcinoma and squamous cell carcinoma are the most common forms of skin cancer, melanoma, the most fatal form of skin cancer, is rapidly increasing in the USA [2]. The American Cancer Society estimated 76,690 new cases of melanoma in 2013 and more than 9,000 deaths will be attributed to this type of cancer during the same year [3]. Ultra violet radiation (UVR) exposure is the greatest modifiable risk factor for the occurrence of all major types of skin cancer, including melanoma [4, 5]. Therefore, to reduce UVR exposure, cancer organizations recommend sun protection practices, including seeking shade, limiting the direct sun exposure in the middle of the day when UVR is at its highest level (between 10:00 a.m. and 4:00 p.m.), wearing sunglasses, covering skin with a protective clothing or hat with wide brim, and using sunscreens with a sun protection factor (SPF) of 30 or higher and a 3- or 4-star UVA protection rating [3, 6].

Outdoor athletes are at an increased risk of skin cancer development because they are routinely exposed to excessive levels of UVR that may be unavoidable during practice and competition [7, 8]. Athletes develop the more common, generally treatable skin cancers and potentially life threatening malignant melanoma [8–10]. Unfortunately, many athletes fail to wear sunscreen because they feel it interferes with their athletic performance; they forget to apply it, or they hope to get a suntan [11, 12]. Furthermore, many sports teams have required uniforms that expose an athlete's face, arms, legs, some portions of the upper and lateral upper back [7]. Long sleeve shirts may restrict the range of motion, and therefore sun protective clothing can become a barrier to performance [13]. Baseball caps, the most common sun protective hats chosen by outdoor athletes, do not provide adequate coverage of the ear, neck, and face [13]. Sunglasses and hats that cover the ears, neck, and face offer greater protection against UVR, but many sports including tennis, field hockey, and soccer prohibit their use [14].

Despite a documented increased risk of skin cancer among outdoor athletes, there is a limited amount of research focused on this group. The purpose of this study was to gather data on collegiate athlete's current knowledge, attitudes, and behaviors regarding risks for developing melanoma and the importance of skin cancer prevention. An additional purpose was to investigate the factors related to the adoption of preventive behaviors. This could support the development of effective strategies for future skin cancer prevention programs targeting this population group.

The study was designed to answer the following research questions.

 (RQ-1) What demographics factors (i.e., age, race, and gender) are correlated with knowledge, attitudes, and behaviors related to skin cancer?
 (RQ-2) Do knowledge and attitudes correlate with behaviors?

The hypotheses for this study were as follows.

 (H-1) There will be no significant correlation of knowledge with age, race, and gender.
 (H-2) There will be no significant correlation of attitudes with age, race, and gender.
 (H-3) There will be no significant correlation of behaviors with age, race, and gender.
 (H-4) There will be no significant correlation between behaviors and knowledge.
 (H-5) There will be no significant correlation between behaviors and attitudes.

## 2. Methods

### 2.1. Sample and Study Design

A nonrandom convenience sample of 393 Division I athletes from the Southern University in the USA was selected for this cross-sectional study. All participants were 18 to 24 years of age.

### 2.2. Data Collection Procedure

The University Institutional Review Board granted permission to conduct this study. Data were collected using a modified version of the Melanoma Risk Behavior Survey (MRBS) [15]. Upon entering the athletic training room or the strength and conditioning facility, participants were offered an opportunity to complete the MRBS. After agreeing to participate, each athlete received an informational letter and the MRBS which required approximately ten minutes to complete.

### 2.3. Instrument

The questionnaire consisted of 55 questions that reviewed demographics, knowledge, attitudes, and behaviors specifically related to the athlete's sun protection and skin cancer risk(s). A total of 24 questions with one correct answer assessed knowledge related to melanoma. Eleven questions assessed attitudes and this section was rated on a five-point Likert-type scale, where one represented strongly disagree and five represented strongly agree. Thirteen questions assessed sun and tanning behaviors. Each possible answer was scored in one of two levels: 50% or more of the time or less than 50% of the time. The coefficient reliability scores for each subscale are knowledge .86, attitude .63, and behavior .92.

### 2.4. Data Analysis

Descriptive statistics were used to depict the characteristics of the study participants. Pearson correlation coefficients were performed to assess the degree of relationships of preventive behaviors with knowledge and attitudes. Furthermore, chi-square analysis was calculated to determine associations of demographics variables (i.e., age, race, and gender) with knowledge, attitudes, and behaviors towards skin cancer. For all analysis, an alpha level was set at .05.

## 3. Results and Discussion

A total of 343 athletes fully completed questionnaires, generating a response rate of 87%. Of these, 54.8% were males and 45.2% were females. The majority of the participants were Caucasian (58.6%) followed by African American (34.4%), Hispanic (1.8%), Asian (0.6%), and Other (4.6%). The age breakdown of the participants was as follows: 18–10.4%, 19–28%, 20–22.3%, 21–17.8%, 22–16.9%, 23–3.2%, and 24–0.3%.  Table 1 presents knowledge, attitude, and behavior by gender, race, and age.

### 3.1. Knowledge Assessment

The total mean score for the knowledge portion was 8.88 ± 4.39, with a total possible score of 24. Knowledge scores are presented in Table 2. This study found that college athletes lacked basic knowledge about skin cancer and sun protection, a finding that is consistent with previous researches conducted with university students [15, 16]. With regard to sunscreen knowledge, 44.9% of the participants knew that individuals should apply sunscreen 15–30 minutes before going outdoors, whereas only 35% recognized that sunscreen should be reapplied every hour. These results suggest that athletic trainers and coaches should provide recommendations for proper sunscreen application and reapplication to their athletes. Though response to knowledge questions demonstrated that athletes are well informed about the risk factors for developing skin cancer, only 20.7% of the athletes knew that working or spending most of the time outdoors is a significant risk factor. This is of concern, considering athletes must know that sun exposure is a primary external cause of melanoma and individuals spending more time outdoors in the direct sunlight could increase their risk of developing skin cancer [17]. Future studies should investigate the extent to which athletes perceive their susceptibility to develop skin cancer due to their occupation.

### 3.2. Attitude Assessment

The mean score was 41.91 ± 6.22, with the total possible score ranging from 11 to 55. This implies a medium level of appropriate attitudes toward sun protection.  Table 3 provides attitude scores. In the present study, 17.5% of participants agreed with the statement “a nice tan improves one's appearance,” and 27.4% agreed that “a nice tan makes one look healthy.” Results from this study are in concordance with previous research documenting that a tanned appearance is considered desirable and attractive [2, 18]. Excessive sun exposure and sun bathing were reported previously among individuals who stated that a suntan is attractive and healthy [18–22]. An unexpected finding emerged from this study was that 83% of participants agreed that “tanning beds improve one's overall health.” It appears that tanning parlors have been successful in promoting the false concept of a safe tan. Athletes should be educated about the hazards inherent in the use of tanning beds to prevent additional UV exposure over and above the amount they cannot avoid during training and competition [23]. Moreover, previous studies supported the efficacy of appearance-emphasized educational interventions in reducing intentions to indoor tanning [24, 25]. There is also a need to educate athletes about positive sun protection behaviors to decrease their risk of developing skin cancer [26].

### 3.3. Behavioral Assessment

The mean behavior score was 3.00 ± 2.70, with the total possible score ranging from 0 to 12. This reveals a low level of sun protection behaviors. Furthermore, as illustrated by Table 4, most athletes do not limit their sun exposure between 10:00 a.m. and 4:00 p.m., and they reported low usage of sun protective clothing. In this study, only 20.7% of participants reported seeing a doctor to have their skin checked for potential cancerous spots or moles. This is important because early detection plays a vital role in the survival rate of melanoma patients. Less than 25% of athletes reported using sunscreen during practice and/or competitions, or at other times when they are outdoors. Increasing the rate of sunscreen use is a critical behavior in this population for any program that hopes to decrease the incidence of melanoma. Sunscreen is a more viable protective measure for athletes who may find it impossible to stay in shade or wear additional protective clothing [10]. It is also reported that it is easier to overcome barriers to sunscreen behavior when compared to barriers that prevent individuals from wearing protective clothing [27].

### 3.4. Hypothesis Testing

There was only one 24-year-old participant in this study. This participant scored very low on the knowledge portion of the questionnaire (2 points out of possible 24) and was therefore eliminated from the analysis for age and knowledge. With the elimination of this participant, our hypothesis was supported. There was no significant association between age and skin cancer knowledge.

Within the narrow age range of 18 to 23 years, scores for the attitude portion of the questionnaire indicate that older athletes demonstrate a better attitude towards skin protection than younger athletes (see Table 1). However, results of chi-square analysis indicated no significant association between age and attitude. Therefore, we fail to reject our hypothesis. This suggests that age does not influence the athlete's attitude regarding skin cancer, its risk factors, and prevention measures.

The behavior scores were low across all age groups (see Table 1). The observed trend is that behavior scores increase slightly up to age 20 years and then begin to decline. The one 24-year-old participant scored the best (4 out of 12 points possible) but was excluded because there was only one participant in this age group. Significance was not achieved between age and skin cancer behaviors, as our hypothesis stated. Therefore, age of the participants did not influence their sun protection behaviors.

Scores for the knowledge portion of the questionnaire indicate that darker-pigmented races are less knowledgeable about skin cancer, its risk factors, and prevention measures (see Table 1). This is not surprising since media and some prevention campaigns tend to target Caucasian audiences. Thus, contrary to our hypothesis, there was a significant relationship between race and knowledge (*χ*2 = 168.08, *P* = .001). Racial factors influence the athlete's knowledge regarding skin cancer, its risk factors, and prevention measures.

Scores for the attitude portion of the questionnaire were low across all racial groups (see Table 1). A trend is observed which suggests that Whites and Hispanics had higher attitude scores, whereas Asians had lower scores. This trend did not reach significance and, therefore, our hypothesis was confirmed. Race does not influence the athlete's attitude regarding skin cancer, its risk factors, and prevention measures.

Scores for the behavior portion of the questionnaire were low across all racial groups, with African Americans and Asians scoring the lowest, respectively (see Table 1). The correlation analysis showed significant association between race and behavior (*χ* ^2^ = 67.78, *P* = .031). We must reject our hypothesis that race does not influence sun protection behaviors.

Females scored higher on the knowledge portion of the questionnaire than their male counterparts (see Table 1). This finding is consistent with the previous studies targeting adolescents and young adults which demonstrated gender differences in skin cancer knowledge, with females reporting higher knowledge than male participants [15, 27, 28]. The results of our data analysis revealed significant association between gender and knowledge (*χ* ^2^ = 48.29, *P* = .001). Hence, the hypothesis was rejected. Gender influenced knowledge of skin cancer, its risk factors, and prevention measures.

Females scored higher on the attitude portion of the questionnaire than their male counterparts (see Table 1). It was found that there was a significant association between gender and attitudes (*χ* ^2^ = 109.48, *P* = .001). Our hypothesis was rejected. The gender of the participants influenced skin protection attitudes.

Consistent with the prior studies [15, 27, 28], females in this study scored higher on the behavior portion of the questionnaire than their male counterparts (see Table 1). There was a significant association between gender and behavior (*χ* ^2^ = 31.18, *P* = .002). Our hypothesis was rejected. The gender, in fact, did influence the participants' sun protection behaviors. Existence of difference between men and women in sun protection could be attributed to women having higher perceived susceptibility to skin cancer and lesser perceived barriers to use of sun protection measures [29]. However, additional study is required that investigates why male athletes resist the adoption of sun protection strategies.

We also investigated the relationship between knowledge and behavior and, in support of our expectations, found a significant positive relationship (*r* = .244, *P* < .05). This information indicates that successful skin cancer educational interventions should focus on athletes' knowledge of their increased risk and methods of sun protection. Further, contrary to our hypothesis, there was no significant relationship between attitude and behavior (*r* = .049, *P* > .05).

## 4. Limitations

There are several potential limitations of the current study. A convenience sample of athletes from only one university was surveyed; thus, caution must be exercised in extending our findings to other universities, especially universities situated in other geographical regions. Also, the majority of the participants in this study were Caucasian; it may not be possible to generalize these results to other ethnic groups. This study results rely on self-reported data, which could introduce recall and social desirability biases. Finally, the results are limited by cross-sectional nature of this study, which means that directions of effects can only be speculated.

## 5. Implications

Athletic departments should provide athletic trainers with funds needed to purchase and dispense sunscreen to the athletes. Athletes participating in outdoor sports with practices and competition times between the hours of 10:00 a.m. and 4:00 p.m. should be reminded by athletic trainers and coaches to reapply sunscreen to avoid both sunburns and chronic sun damage. Athletes should be educated by their athletic training and coaching staff(s) on the correct way to perform skin self-examination, and they should be encouraged to see a physician for any changing or abnormal moles. Athletic directors, athletic trainers, coaches, and other individuals involved in the health and safety of college athletes may also need to be educated to include sun safety measures in their discussions with the athletes during meetings and practice sessions. Lastly, athletes should be educated on the hazards of indoor tanning facilities as well as encouraged to avoid using these facilities.

## Figures and Tables

**Table 1:** Knowledge, attitude, and behavior by gender, age, and race.

	Knowledge		Attitude		Behavior	
	(24 points possible)		(55 points possible)		(12 points possible)	
	M	SD	M	SD	M	SD
Gender						
Male	7.60	4.54	39.06	5.93	2.69	2.72
Female	10.43	3.67	45.36	4.62	3.37	2.63
Age (years)						
18	8.28	4.38	42.42	5.44	1.94	1.99
19	9.02	4.00	41.55	6.00	3.09	2.64
20	8.61	5.01	41.49	6.78	3.76	3.08
21	9.08	4.17	41.85	6.59	3.34	2.97
22	9.36	4.27	42.64	6.06	2.17	1.85
23	8.55	5.11	42.27	5.76	2.36	2.69
Race						
Caucasian	10.29	3.77	43.24	5.48	3.41	2.75
African American	6.66	4.00	39.67	6.98	2.26	2.48
Hispanic	11.50	5.21	43.83	5.67	4.33	2.16
Asian	7.00	4.24	38.00	2.83	2.50	2.12
Other	6.75	6.48	41.44	4.91	2.81	2.74

**Table 2:** Correct responses to knowledge questions.

Questions	% Correct response
The most common form of skin cancer is?	5.2% (basal cell carcinoma)
Women primarily develop melanoma on what area of their body?	11.4% (the legs)
Men primarily develop melanoma on what area of their body?	20.7% (the back)
The risk that an American will be diagnosed with melanoma is?	22.4% (one in 55)
Which of the following statements is true?	51.6% (African Americans and those with darker skin can contract skin cancer)
Which of the following is not a recommended way to reduce skin cancer risk?	29.7% (drink plenty of noncarbonated fluids)
The most common treatment for melanoma is?	33.8% (surgery)
Early cases of melanoma are almost what percentage (%) curable by proper surgical procedures?	12.8% (100%)
The melanin in African American's skin filters out how much UVB rays as compared to the melanin in Caucasian's skin.	20.7% (two times more)
The prognosis for African American's once they are diagnosed with skin cancer is?	17.8% (worse than Caucasians)
Which of the following is not a sign of melanoma?	38.8% (the color over the entire mole is the same)
The main source of UV radiation is?	68.5% (sunlight)
The risk of melanoma is greater if how many of a person's first-degree relatives have been diagnosed with melanoma?	10.8% (one or more)
What is the recommended amount of time that individuals should apply sunscreen before going outdoors?	44.9% (15–30 minutes)
How often should individuals reapply sunscreen?	35% (every hour)
The proper amount of sunscreen to fully cover a human body would be equivalent to?	23.3% (a shot glass full)
Risk factor knowledge questions	
Having dark colored skin?	58.3% (no)
The number or type of moles on the body?	58% (yes)
Having dark colored skin?	58.3% (no)
Having black or dark brown hair?	58.3% (no)
Having red or blonde hair?	41.7% (yes)
Having freckles?	54.8% (yes)
Overexposure to the sun or UV radiation when a child?	75.5% (yes)
A family history of skin cancer?	73.2% (yes)
Individuals that work or spend most of their time outdoors?	20.7% (yes)

**Table 3:** Correct responses to attitude questions.

Questions	% Correct response
Tanning beds improve one's overall health	2.3% (strongly disagree)
The use of sunscreen will help to protect me from getting melanoma	47.2% (strongly agree)
A nice tan improves one's appearance	25.4% (strongly disagree)
Examinations by a dermatologist help to detect the early stages of melanoma	48.1% (strongly agree)
A nice tan makes one look healthy	12.2% (strongly disagree)
Self-checks for melanoma help to detect the early signs of melanoma	37.6% (strongly agree)
A good tan is worth the increased risk of skin cancer	42.6% (strongly disagree)
Public awareness about melanoma is important to reduce risk	47.5% (strongly agree)
Only fair skinned people need to be worried about sun exposure	5% (strongly disagree)
All people should take precautions against the damaging effects of the sun	56% (strongly agree)
I believe I should practice sun safe behaviors	46.1% (strongly agree)

**Table 4:** Correct responses to behavior questions.

Questions	% Correct response
How often do you attempt to avoid the sun between the hours of 10 a.m. to 4 p.m. in order to reduce sun damage excluding practice/competitions?	14.9% (more than 50% of the time)
How often does your sport practice/compete outside during the hours of 10 a.m. to 4 p.m.?	38.8% (50% or less of the time)
How often do you attempt to cover up with tightly woven clothes and hats to avoid the sun excluding practice/competitions?	12.2% (more than 50% of the time)
How often do you see a doctor to have he/she check your skin for potential cancerous spots or moles?	20.7% (more than 50% of the time)
Do you use tanning beds?	67.6% (no)
How often do you regularly use sunscreen when exposed to the sun excluding practices/competitions?	24.2% (more than 50% of the time)
How often do you regularly use sunscreen when exposed to the sun during practices/competitions?	23% (more than 50% of the time)
How often do you use the equivalent of a shot glass of sunscreen each time you apply it during non-practice/competition times?	17.8% (more than 50% of the time)
How often do you use the equivalent of a shot glass of sunscreen each time you apply it before practice/competitions?	12.8% (more than 50% of the time)
How often do you reapply sunscreen every three hours when in the sun during non-practice/competition times?	16.9% (more than 50% of the time)
How often do you reapply sunscreen every three hours when in the sun during practice/competitions?	13.7% (more than 50% of the time)
How often do you reapply sunscreen after being in the water?	15.7% (more than 50% of the time)
How often do you use a sunscreen with a SPF of 15 or higher?	34.1% (more than 50% of the time)
